# Pilot feasibility study to determine the utility of direct access and quantitative magnetic resonance cholangiopancreatography (MRCP) in the assessment of suspected acute biliary or ductal gallstone presentations

**DOI:** 10.1186/s12876-025-03637-0

**Published:** 2025-02-10

**Authors:** Alex Novak, Anita Acharya, Sally Beer, Alexis Espinosa, Giles Bond Smith, Cyrene Saga, Jane Andrews, Adam Bailey, Zahir Soonawalla, Helen Bungay, Michael Pavlides

**Affiliations:** 1 Emergency Medicine Research Oxford (EMROx), Oxford University Hospitals NHS Foundation Trust, Oxford, UK; 2 Oxford University Hospitals NHS Foundation, Oxford, UK; 3 Emergency Medicine Research in Oxford, Oxford, UK; 4 Oxford University Hospitals NHS Foundation, Oxford, UK; 5 Oxford University Hospitals NHS Foundation Trust, Oxford, UK; 6 Head of Liver Imaging Research, Oxford Centre for Clinical Magnetic Resonance Research (OCMR), Radcliffe Department of Medicine, University of Oxford, Oxford, UK; 7 Emergency Department, John Radcliffe Hospital, HeadleyWay, Headington, Oxford OX39DU UK

**Keywords:** MRCP (Magnetic Resonance Cholangiopancreatography), Gallstone disease, Diagnostic pathways, Healthcare costs, AI-assisted imaging analysis

## Abstract

**Background:**

Patients with suspected acute gallstone disease typically undergo abdominal ultrasound. MRCP is often used for patients with abnormal LFTs, potentially making ultrasound unnecessary for this group. Despite high inter-reader variability in MRCP interpretation, new AI technologies may automate and standardize detection and measurement.

**Method:**

Patients with suspected acute gallstone disease and abnormal liver function tests were randomized into two diagnostic pathways, direct MRCP and standard care. Admission data, healthcare resource use, and clinical outcomes were recorded. National Health Service national 20/21 tariffs were used to calculate and compare healthcare costs. MRCP scans were subsequently analysed using MRCP + software (Perspectum Ltd).

**Results:**

27 participants were enrolled over 12 months, 15 to direct MRCP and 11 to standard care. One patient was excluded from analysis. Mean patient time to diagnostic report and mean per patient associated direct medical cost and mean cost to diagnosis for the direct MRCP and standard of care group was 2.53 days, £449.54, and £647 respectively for the direct MRCP group and 4.18 days costing £742.06 and £896 for standard care. MRCP + analysis of 11 scans showed significant differences between the groups in terms of gallbladder volume (80.2mm^3^ gallstone present versus 30.1mm^3^ without, *p* = 0.018 and cystic duct median width (4.6 mm gallstone present versus 2.7 mm without, *p* = 0.042).

**Conclusions:**

Direct MRCP may be a feasible and potentially cost-effective diagnostic strategy for patients with suspected acute gallstone disease and deranged LFTs. Automated measurement of MRCP parameters shows promise in detecting obstruction. Larger trials are warranted to assess this potential.

**Clinical trial number:**

This study is registered with ClinicalTrials.gov (NCT03709030). Registration date: October 17, 2018.

**Supplementary Information:**

The online version contains supplementary material available at 10.1186/s12876-025-03637-0.

## Background

Acute gallstone disease comprises a significant proportion of surgical admissions and presentations to Emergency Departments (EDs) [1–3]. Patients with suspected acute gallstone disease are usually clinically assessed and investigated with blood tests including Liver Function Tests, with subsequent imaging typically including initial abdominal ultrasound (US) to confirm the presence of gallstones and identify features that may suggest cholecystitis or the presence of bile duct stones [4]. Magnetic resonance cholangiopancreatography (MRCP) is recommended if ultrasound has not detected ductal stones but the bile duct is dilated and/or liver function test results are abnormal, with endoscopic ultrasound (EUS) typically reserved for patients in whom MRCP does not allow for a diagnosis to be made [5]. In practice MRCP is frequently requested along with US in the work-up of suspected gallstone patients, has been shown to be more cost-effective than US in selecting patients for invasive interventions like Endoscopic Retrograde Cholangio-Pancreatography (ERCP) with suspected CBD stones [6]. Furthermore, MRCP has a comparable accuracy to ERCP [7], and has been shown to reduce the number of unnecessary procedures performed in gallstone patients [8]. 

There is often considerable delay in carrying out two sequential imaging investigations for each patient, resulting in an increased length of stay and increasing bed occupancy [9]. Generally emergency departments have limited access to MRCP, which is usually performed as part of a surgical admission or outpatient investigation. Several studies have also raised questions regarding the utility of ultrasound in acute biliary disease, with concerns raised around limited sensitivity and user dependence [10–12]. Retrospective studies have supported the use of MRCP as a diagnostic test over US and suggested the potential for economic benefits in patients with potential choledocholithiasis [13, 14]. Direct use of MRCP as a first line imaging modality may therefore be cost-effective compared with existing diagnostic strategies in these patients [15]. 

Currently interpretation of MRCPs is a qualitative manual assessment, which can lead to significant inter-reader variation [5, 16, 17]. Recent advances in image processing techniques have enabled artificial intelligence (AI)-driven quantification of the biliary tree using quantitative MRCP. This has been shown to improve the assessment of patients with biliary diseases in outpatient settings with improved prognostic capabilities or detection of progressing disease than traditional radiology reads in patients with Primary Sclerosing Cholangitis (PSC) [18]. However, to date this technique has not previously been applied in an acute setting in patients with suspected obstructive gallstone disease.

The aim of the Early MRCP in Acute GallstonE Disease (E-MAGED) pilot study was to compare the potential impact on diagnostic efficiency and cost-effectiveness, and overall feasibility and utility of employing a diagnostic strategy of direct MRCP versus standard care (USS +/- MRCP or other imaging) in patients presenting as emergency admissions to a UK teaching hospital with suspected acute gallstone disease. As quantitative MRI techniques can potentially provide clinically useful information not currently provided by current imaging techniques used in clinical practice, a further exploratory aim was the evaluation of the feasibility and utility of employing quantitative MRCP in the assessment on biliary dilatation secondary to acute obstructive gallstone disease.

## Methods

**Fig. 1:**
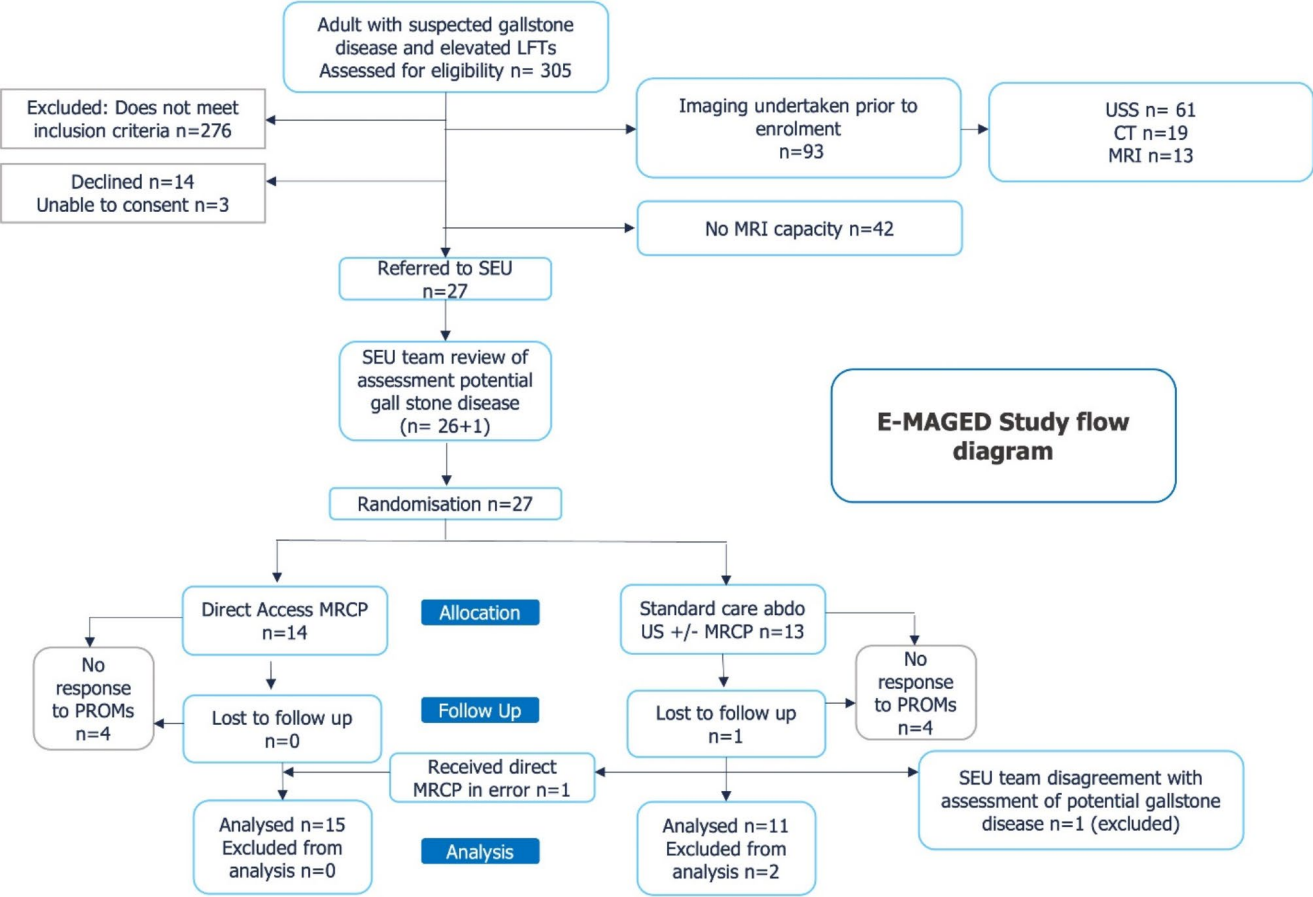
Study flow chart

This protocol follows the CONSORT 2010 guidelines for reporting randomized controlled trials (Please see Fig. 1).

### Study design and participants

We undertook a pilot prospective randomised, controlled, single-centre superiority feasibility trial with two parallel arms which compared two diagnostic strategies: direct MRCP versus standard care. Adult patients presenting to the John Radcliffe Hospital (Oxford, UK) Emergency Department, Surgical Emergency Unit or Ambulator Assessment Unit with acute abdominal pain were eligible for recruitment if they had at least one deranged liver function test (see Table 1), and clinically suspected gallstone/biliary disease. A full summary of inclusion and exclusion criteria is summarised in Table 1.

**Table 1:**
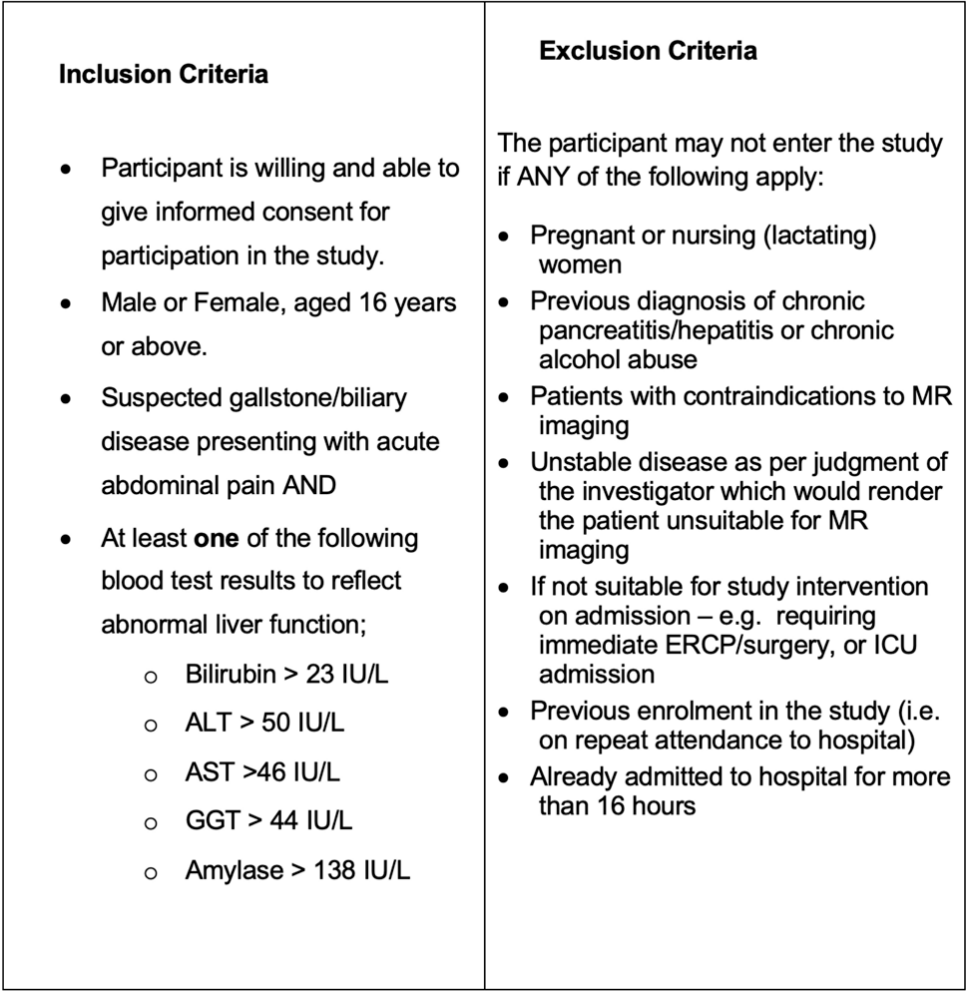
Inclusion and exclusion criteria for enrolment, including thresholds for biochemical derangement

### Procedures

Patients presenting to the ED, AAU or SEU were screened and identified by ED research nurses based on their clinical presentations and blood results. Computerised randomisation was performed following a minimisation design with a 1:1 allocation. Study recruits were randomly allocated to one of two diagnostic pathways: (a) direct MRCP within 16 h of initial attendance (MRCP used as the initial mode of imaging), or (b) standard care (abdominal ultrasound, with further imaging as deemed appropriate by the responsible clinical team). The 16 h cut-off for direct MRCP was decided upon based on the practical considerations of scheduling MRCP as part of an acute care pathway. Local radiology demands and pathways necessitated a minimum 8 h to schedule the MRCP scan, and it was felt feasible and necessary to aim for all first line imaging for patients within the study to be undertaken within 24 h. Convenience sampling was undertaken based on both research nurse availability and confirmed scanner availability prior to enrolling patients. If identified in the ED or AAU, participants subsequently had responsibility for their clinical care transferred to the on-call surgical team based at the SEU as per normal clinical practice. These patients were further assessed by the surgical team, who confirmed or disagreed with the clinical suspicion of gallstone disease. If the surgical team confirmed the clinical suspicion of gallstone disease, patients were then consented and randomised, and the relevant scans requested. If the clinical suspicion of gallstone disease was disputed, the patients were excluded from the study prior to enrolment and randomisation, and the surgical team managed and investigated the participant as they deemed appropriate. Enrolled participants were followed up by the research team, and clinical and imaging data retrieved from the EPR and documented on a daily basis until discharge, whereafter they received a three-month telephone follow-up to identify any subsequent readmissions/adverse events and complete the Patient Reported Outcome Measures (PROMs) questionnaire.

All imaging was performed using standard clinical pathways and protocols. MRCP was performed by clinical radiographers as per standard clinical practice via a General Electric (GE) 3T Scanner. Inferencing of images using MRCP + took place downstream from the acute clinical pathway and was not available to inform acute clinical management.

### Health economic methodology

Health economic metrics included time to diagnosis (calculated as days from hospital presentation until receipt of the diagnostic report, i.e. the scan report which took place prior either to intervention or discharge, regardless of modality), overall patient length of stay between presentation and discharge, and proportion of patients with a definitive diagnosis (defined as report ruling in or out the presence of gallstones, i.e. choledocholithiasis or cholecystolithiasis). Patient level data were obtained through hospital personnel and electronic patient record data and analysed in Excel. The study quantified and comparatively analysed the type, frequency and overall costs associated with diagnostic imaging. Direct medical costs associated with imaging, patient length of stay, complications and readmissions are included in the analysis. Capital costs associated with ultrasound and MRCP, as well ED related overhead charges are excluded from the analysis.

To value unit health care resource use, NHS national tariffs (unbundled), derived from the 2020/21 National Tariff Payment System (price payable by commissioners for NHS services) were applied to ultrasound imaging and Magnetic Resonance Imaging (MRI) for MRCP. Costs associated with patient length of stay were also derived from the 2020/21 National Tariff Payment System, namely the HRG “non-malignant, hepatobiliary or pancreatic disorder, without interventions, with Complexity and Comorbidity (CC) Score 0–1”. To calculate a daily tariff rate to facilitate cost of diagnosis (sum of imaging plus bed stay duration until receipt of final diagnostic report) the HRG tariff was divided by length of stay until report received. It is recognized that real world patient resource use will vary depending on patient CC score. The trim point (excess bed day payment or a long stay payment) for the above HRG was included. For patients waiting beyond the trim point (12 days) the excess bed day payment was applied. It is recognized that in the real world the trim point will also vary depending on patient complexity and comorbidity. To account for cost uncertainty and its association with patient case-mix in a larger cohort, a minimum and maximum daily tariff was included in an uncertainty analysis, carried out to approximate the annual cost of direct MRCP for OUH NHS FT. It is noted that national prices are adjusted by national variations such as the market forces factor (MFF) before payment is made to the NHS service. Adjustment for differential timing did not occur as time horizon did not go beyond 12 months.

### Outcomes

#### Primary outcome

The primary outcome measure was the average time to diagnosis, defined as the time measured from hospital attendance to the time of the final imaging report prior to surgical/procedural intervention or discharge from hospital.

#### Secondary outcomes

Secondary outcome measures were used to characterize any wider impact the intervention may have had on the patient’s journey in hospital by undertaking a cost consequence analysis. These measures sought to address the overall ‘utility’ of direct MRCP through assessment of the: (a) overall length of stay, (b) cost of admission, (c) cost of investigation, (d) time to diagnosis, (e) in-hospital complications, (f) re-admission and re-attendance rates, and (g) PROMs.

#### Exploratory outcome

MRCP examinations were post-processed using MRCP+ (Perspectum Ltd, Oxford, UK) to provide quantitative models of the biliary tree. The ability of quantitative MRCP metrics to distinguish patients with biliary dilatation secondary to gallstones from those without was assessed.

### Data and statistical analysis

A priori sample size calculations were performed using preliminary audit data of patients with a documented diagnosis of acute gallstone disease in the ED. We calculated that 27 participants were needed in each parallel arm (54 participants in total) to provide 80% power at the 5% significance level. Using these parameters, 64 participants were anticipated for the study, accounting for a maximum 20% attrition rate. The predicted study duration required to achieve recruitment targets was 12 consecutive months.

For the primary outcome, an independent two-sample t-test was used to analyse the difference in mean time to diagnosis between standard care and direct MRCP groups. Whilst for the secondary outcome, independent two-sample t-tests were used to analyse the difference in mean cost of admission, cost of investigation, time to diagnosis, length of stay and PROM between standard care and direct MRCP groups. For the exploratory outcome analyses, measurements of total volume of the biliary tree and maximum diameter of the common bile duct (obtained from MRCP+) were compared to previously published upper limits of normal [19]. 

Chi-squared tests were used to test for differences in readmission/re-attendance rates and complication rates between the standard care and direct MRCP groups. Data are presented as mean and standard deviation or median as specified and p-values < 0.05 were taken to imply statistical significance.

## Results

Between July 2019 and October 2020, 256 patients were screened, and 27 participants were recruited. A table of the screening log is presented in the Table 1 in supplementary materials. A total of 14 participants were randomly assigned to direct MRCP and 13 participants assigned to standard care. Study delivery was significantly affected during the coronavirus disease 2019 (COVID-19) pandemic due to redeployment of research staff and halting of all non-COVID research activity within the hospital. Recruitment was paused for over 6 months during the trial period, and following interim analysis trial recruitment was later terminated at 27 participants in total, 18 months after the study’s initiation. All participants had daily follow-up throughout their admission and were contacted by phone for follow up at three months post-recruitment. No participants formally withdrew from the trial following recruitment.

A study flow diagram is featured below (see Fig. 1). There were two protocol deviations due to errors by the research delivery team. In one deviation, the delivery team omitted to undertake the extra participant eligibility check with the SEU team as per the protocol, leading to a patient being randomised to the standard care are arm in whom the differential diagnosis did not include gallstone disease. This was identified shortly after randomisation and before the participant received any further clinical care. Following review with the trial steering team it was deemed appropriate to exclude this patient from analysis, as they did not subsequently undergo either the standard care or direct MRCP diagnostic pathways. In the second protocol deviation, one of the patients randomised to standard care received intervention as per the direct MRCP arm of the trial. Two further participants, randomly assigned to the direct MRCP group, were unable to tolerate the MRI procedure: one participant was discharged, whereas the other went on to have alternative imaging. Both were included in the direct MRCP arm for analysis. There were no serious adverse events reported during the trial.

Of the 15 participants analysed in the direct MRCP arm, 10 were diagnosed as having gallstones, with 3 having common bile duct stones. In the standard care arm, 7 patients were diagnosed with gallstones, 2 with common bile duct stones. The baseline characteristics between study groups are summarised in Supplementary Table 2. Most participants were female (69%), presenting with abdominal pain as their primary symptom and attended hospital initially via the ED (77%). The mean age of study participants was 46.6 years.

In terms of the imaging pathway in standard of care arm, the treating physician ordered ultrasound of duration less than 20 min without contrast for 11 (100%) participants. In addition, for one participant, prior to diagnosis ultrasound was requested twice. To further aid clinical decision making in the standard of care arm, the treating physician ordered MRI scan of one area, without contrast for 7 (63.6%) standard of care assigned patients, and for 2 (18.1%) of these participants, MRI was ordered twice.

In the direct MRCP arm, the treating physician ordered MRCP for all 15 (100%) participants. Median costs of imaging per patient were lower in the direct MRCP group (£108) as compared to the standard care group (£147). Table 2 summarises the type, unit tariff and frequency of the different types of imaging recorded in the two groups.

**Table 2:**
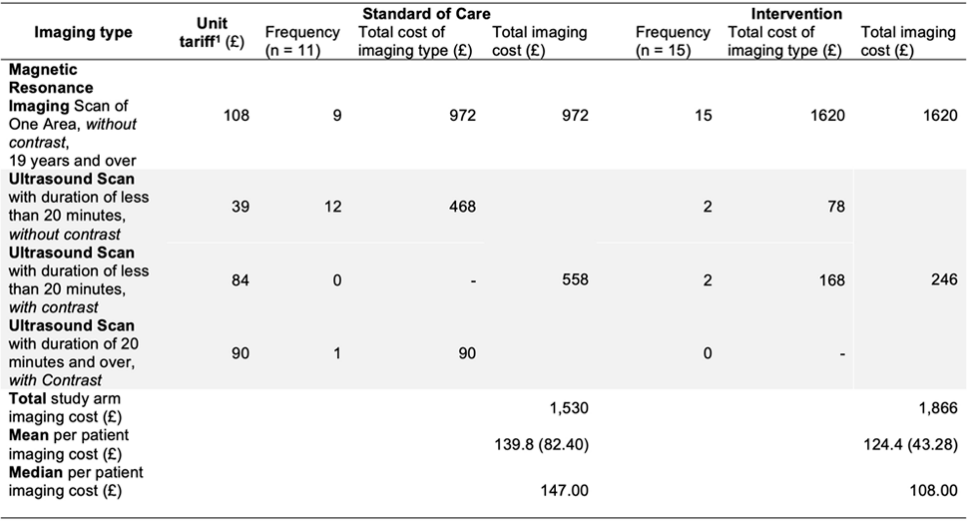
Summary of imaging test by group: type and associated reimbursement cost

In terms of time to diagnosis, defined as duration in days from presentation until receipt of final diagnostic report, the direct MRCP group participants experienced a shorter mean time to diagnostic report coupled with a lower, mean inpatient bed day total cost. The mean patient time to diagnostic report and mean per patient associated direct medical cost for the direct MRCP and standard of care group was 2.53 days, costing £449.54 and 4.18 days costing £742.06 respectively (see Table 3).

**Table 3:**
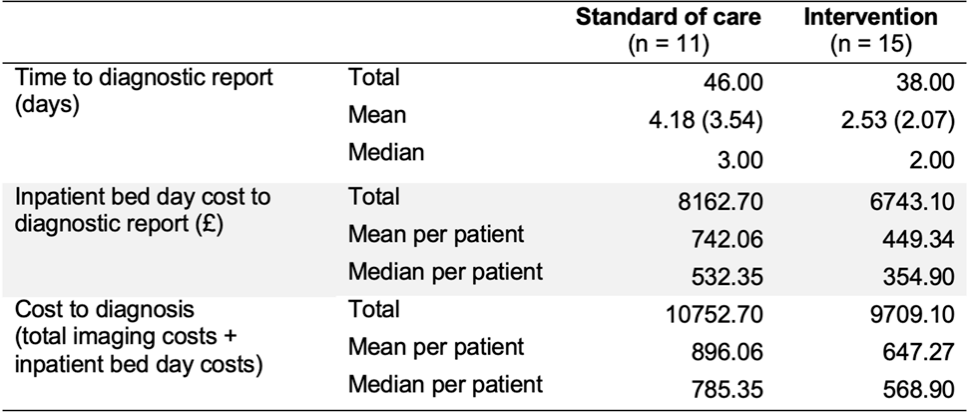
Inpatient length of stay until diagnostic scan report and associated costs: group comparison

The mean diagnosis cost for each group, defined as the sum of imaging plus inpatient bed days until receipt of diagnostic report, for the direct MRCP and standard of care groups was £647 and £896 respectively.

We estimate that in relation to annual incident rates and extrapolating study findings to the hospital Trust, around one patient per day presents to the ED and a similar number presenting to the Surgical Unit via GP referral. Therefore, annually there are an estimated 730 eligible patients for direct MRCP. Based on the mean per patient cost to diagnosis outlined in Table 3, an estimated cost saving of £229,220 is forecast should direct MRCP be implemented rather than standard of care.

Seventeen patients (63%) completed patient reported outcome questionnaires. All patients who completed questionnaires reported abdominal pain at enrolment into the study. No significant differences were observed in between patients in the standard of care or intervention arms in the other types of symptoms reported (*P* > 0.26), duration of symptoms had been occurring for (*P* = 0.21), impact of that the symptoms were having on the patient (*P* = 0.47), or the symptoms reported severity (*P* = 0.54) as shown in Supplementary Table 3. Patients in the intervention arm reported their symptoms had been occurred less frequently than those in the standard of care arm (two patients [22%] in the intervention arm reported symptoms occurring weekly or daily compared to six patients in the standard of care arm [75%], *P* = 0.04).

Overall, patients were very satisfied or satisfied with their level of care with no significant differences were observed in patient satisfaction between the standard of care or intervention arms (Supplementary Table 4). During the 3-month follow-up four patients who completed a PROMs questionnaire reported attending a General Practitioner appointment or presenting at hospital and there were no significant differences between the standard of care or intervention arms (Supplementary Table 5).

11 patients had MRCP scans which were quantifiable, the remainder were excluded due to reduced image quality. Eight (73%) of these patients were diagnosed with gallstones, with the remaining diagnosed with pancreatitis and deranged LFTs of uncertain aetiology respectively. A summary of the groupwise differences in measurements between gallstone and non-gallstone groups is presented in Table 4 and supplementary Fig. 1.

**Table 4:**
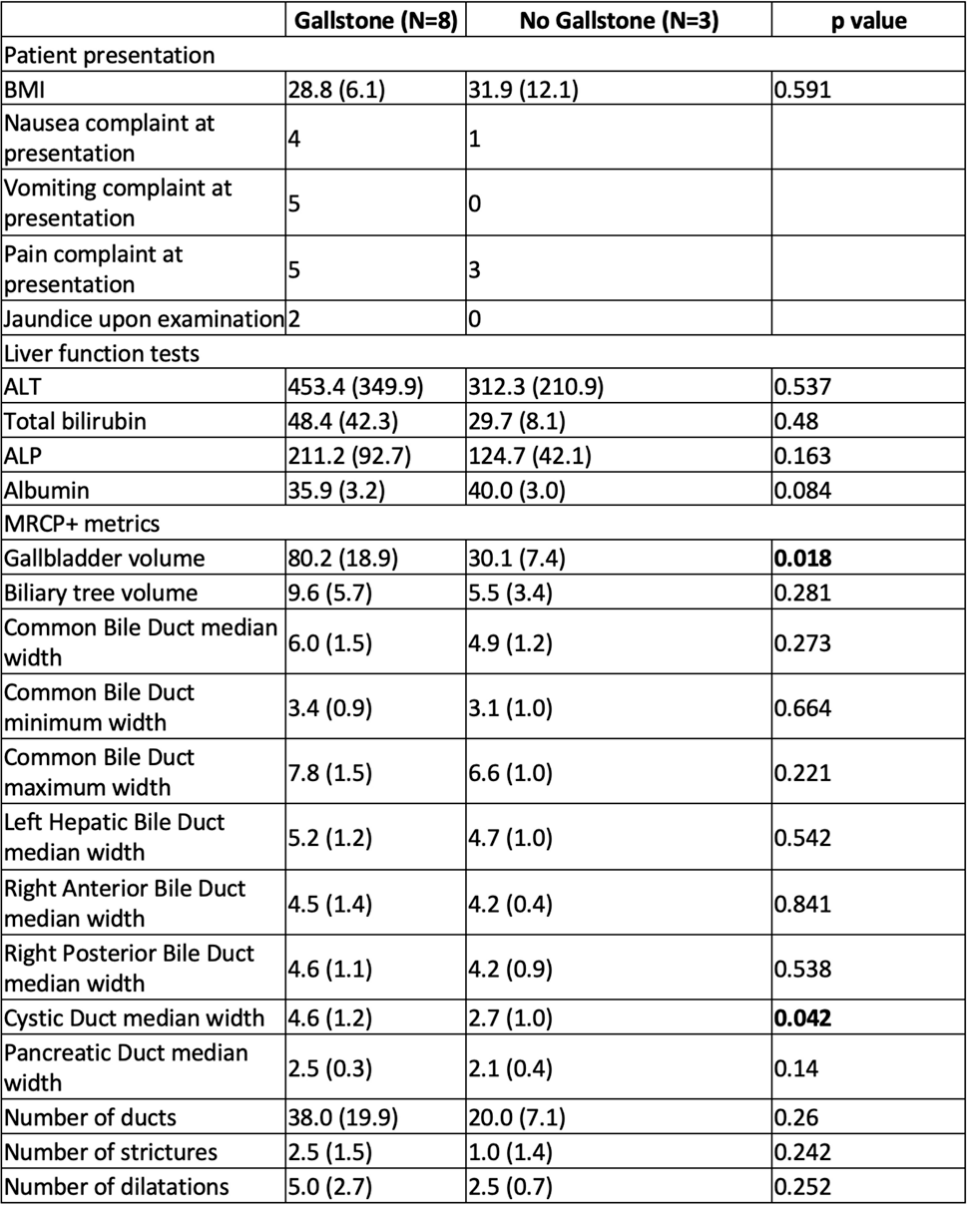
Summary of patient presentation, liver function tests and MRCP + metrics between those with and without gallstones (independent t-test used to investigate group differences)

**Fig. 2:**
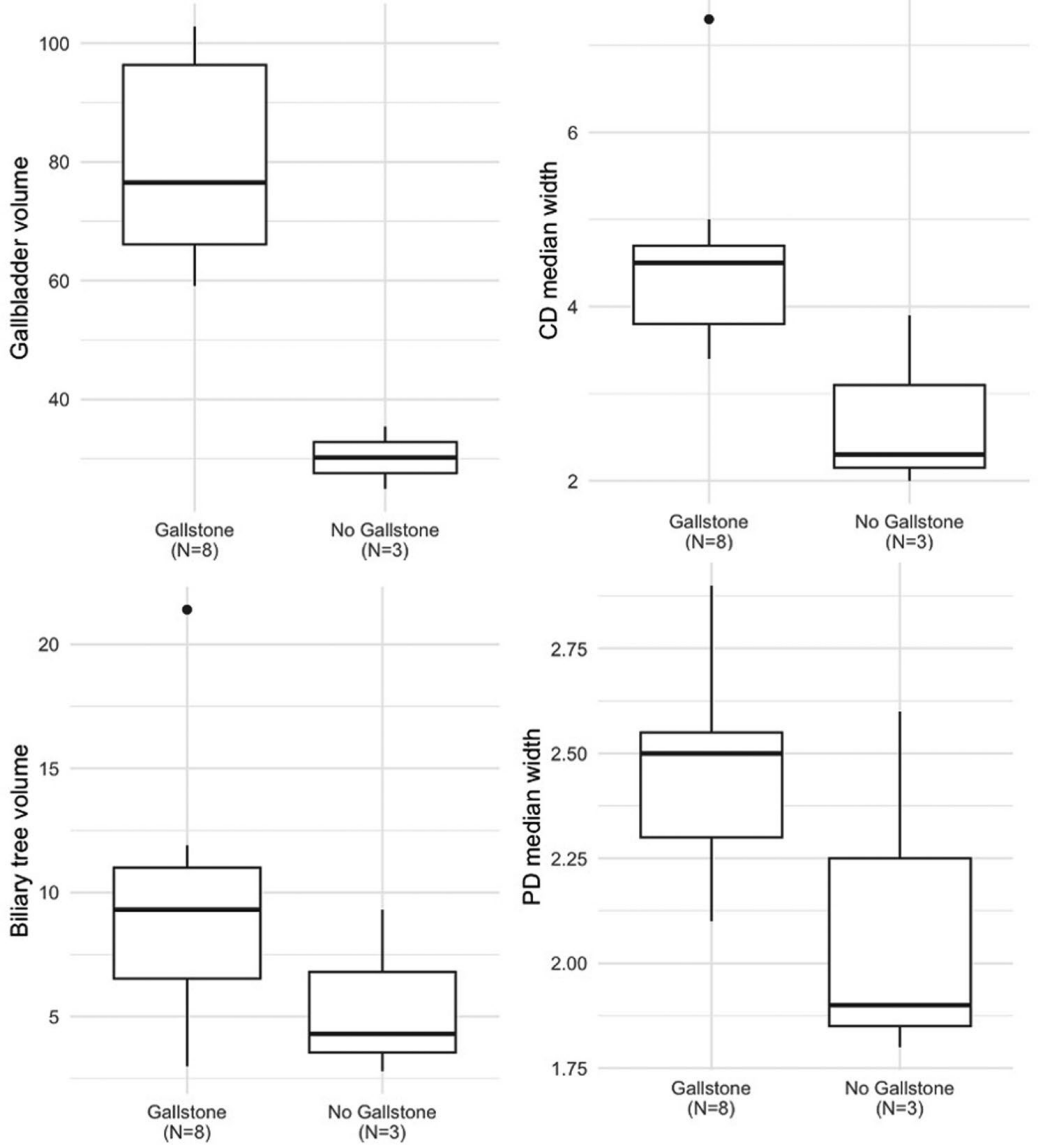
Groupwise differences in MRCP + metrics between those with and without gallstones for Gallbladder Volume, Cystic Duct Median Width, Biliary tree Volume, Pancreatic Duct Median Width. Significant differences were seen in gallbladder volume and cystic duct median width measurements between the groups

Figure 2 shows the groupwise differences in patients. Most patients with gallstones (75%) had elevated total biliary volume compared to established upper limits of normal (biliary volume = 9.3 mL [6.5–11 mL]). However, only four (50%) patients with gallstones demonstrated dilation of the common bile ducts (maximum common bile duct diameter = 7.4 mm [7.0–8.1 mm]). A comparison of diagnostic accuracy of quantitative MRCP compared to traditional MRCP was not performed due to the early termination of the study and corresponding reduced sample size. Example patients who were suspected of having acute gallstone disease but later shown to have an alternate cause, stones within the gallbladder and a gallstone within the common bile duct are shown in Supplementary Fig. 2.

## Discussion

This study, which included 27 patients with suspected acute gallstone disease presenting to acute hospital services demonstrated a reduced time to diagnosis, overall length of stay, cost to diagnosis and overall imaging cost in the direct MRCP group compared with the standard care group. The primary outcome of time to diagnosis was significantly reduced in the direct MRCP group (2.53 days in the direct MRCP group 4.18 days in the standard care group), with a lower mean cost of direct care (£449 versus £742) and lower total cost to diagnosis (£647 versus £896).

The standard care and direct MRCP groups were balanced in terms of age, gender, diagnosis, and presence of gallstones within the constraints of a small sample size – the slightly increased female preponderance in the study population as a whole is commensurate with a higher prevalence of gallstone in women. There was an overall high prevalence of gallstones in the enrolled group (19 out of a total 27 participants), with a high level of concordance between the clinical assessments made by the surgical and ED teams clinical assessments (disagreement in only 1 patient out of 27), suggest that the clinical judgement of ED clinicians was sufficiently reliable in detecting the presence of gallstone disease in this patient population to support the study.

The study also demonstrated the feasibility of performing quantitative MRCP assessments in an acute setting and identified increased dilatation of the CBD and whole biliary tree in patients with gallstone disease compared to normal biliary trees. Gallbladder volume and cystic duct width measurements were significantly different between groups, and biliary volume and pancreatic duct median width were elevated in the gallstone group. The numbers in this analysis were small however in part due to variable image quality, thus limiting the strength of any conclusions, and the impact of this additional information on clinical judgements, time to diagnosis, and cost of diagnosis for patients with suspected gallstone disease should be examined in future studies.

There have been few studies to date which directly compare diagnostic strategies for suspected acute gallstone disease. Milburn et al. conducted a retrospective study examining the patient journeys corresponding to 234 inpatient MRCP scans over a 2-year period. They found that increasing access to MRCP led to further interventions during that admission in 22% of cases due to an increased detection of significant complications and alternate pathologies (e.g. malignancy), and therefore increased overall median length of stay to 3 days from request to report [20]. Recruitment is ongoing for a multi-centre pragmatic randomised controlled trial (RCT), the Sunflower Study, which aims to evaluate the efficacy and cost-effectiveness of pre-operative MRCP for the management of symptomatic gallstone disease [21]. The overall prevalence of CBD stones in the group (5 out of 27, 18.5%) was high in comparison to other similar studies, an anticipated result of restricting enrolment to only patients with deranged LFTs in our study.

This study used a pragmatic approach based on existing care pathways at a large teaching hospital to explore the feasibility of conducting a definitive trial comparing diagnostic strategies in the assessment of suspected acute gallstone disease. The study participants were fully randomised to include a control group which accurately reflected the variety of diagnostic strategies utilised in routine care and were followed throughout the duration of their acute admission, considering all associated costs in the analysis. As such the results of this evaluation reflect a pragmatic assessment of the health economic implications of this approach, which supports their validity and generalisability. This study also represents the first evaluation of a novel technology with the potential to automate and standardise MRCP measurements and demonstrated an early positive signal in this regard.

In terms of limitations, this pilot feasibility study recruited only small numbers of participants across a prolonged timeframe. Recruitment for this study was significantly lower than expected, due to several challenges encountered by the study team, including a relatively limited availability of MRI slots following reduced capacity due to the breakdown and subsequent replacement of MRI scanners, compounded by disruption due to the impact of the COVID-19 pandemic on clinical and research services during that period, and the number and nature of patients presenting to hospital. These interruptions in recruitment limit the representativeness of the sample, and consequently the generalisability of the results. Access to MRI scanner slots was delivered by using fixed slot times, which in addition to restrictions on available research nurse time markedly reduced the time window for recruitment of participants – the limitation on scanner availability may be significant in smaller centres with more limited radiology capacity. Two patients in this study were unable to tolerate MRCP due to claustrophobia/discomfort, and this could potentially limit the efficacy of a direct MRCP diagnostic strategy, though ultrasound also has known failure rate, e.g. pain on performing procedure, difficulty obtaining images – neither of these failure rates were characterised in this study and would need to be considered in future definitive studies. The increasing implementation of ambulatory pathways in line with Same Day Emergency Care may also alter the cost implications for different diagnostic strategies – this aspect was not explored in this study. Additionally, the relatively small number of patients recruited, and scans subsequently inferenced by MRCP + prevented a definitive comparison of the diagnostic accuracy of novel quantitative MRCP assessments to the traditional qualitative MRCP reads in this setting, or any effect on patient outcomes. These important considerations should be included in larger more definitive studies as part of any future evaluation of the technology.

## Conclusion

It may be feasible to use MRCP over abdominal ultrasound as a first line diagnostic test in suspected acute gallstone disease with deranged liver function tests, and this alternative diagnostic strategy may prove cost effective overall in reducing imaging costs and length of stay. Artificial Intelligence-assisted MRCP measurement is feasible in the acute setting and may facilitate automation and standardisation. Larger studies are required to fully explore the potential of these findings.

## Electronic supplementary material

Below is the link to the electronic supplementary material.

Supplementary Material 1

## Data Availability

All datasets and documents related to this study currently reside securely in Oxford University Hospitals NHS Foundation Trust, and will be made available upon reasonable request to the corresponding author.

## Declarations

### Guarantor

The guarantor of the study is Prof Alex Novak; He accepts full responsibility for the finished work and/or the conduct of the study, had access to the data, and controlled the decision to publish.

### Transparency declaration

The lead author, Prof Alex Novak, affirms that this manuscript is an honest, accurate, and transparent account of the study being reported; that no important aspects of the study have been omitted; and that any discrepancies from the study as planned have been explained.
