# Supplementary material for: Pilot feasibility study to determine the utility of direct access and quantitative magnetic resonance cholangiopancreatography (MRCP) in the assessment of suspected acute biliary or ductal gallstone presentations
Source: BMC Gastroenterol. 2025 Feb 10;25:72. doi: 10.1186/s12876-025-03637-0 (PMC11809031; doi:10.1186/s12876-025-03637-0)

**Pilot feasibility study to determine the utility of direct access and quantitative magnetic resonance cholangiopancreatography (MRCP) in the assessment of suspected acute biliary or ductal gallstone presentations.**

| **Authors:** | **Professor Alex Novak**, Consultant in Emergency Medicine and Ambulatory Care, Emergency Medicine Research Oxford (EMROx), Oxford University Hospitals NHS Foundation Trust, alex.novak@ouh.nhs.uk, https://orcid.org/0000-0002-5091-6768 |
| --- | --- |
|  | **Anita Acharya**, Oxford University Hospitals NHS Foundation, anita.acharya@ouh.nhs.uk  **Sally Beer**, Lead Research Nurse for Urgent and Emergency, Emergency Medicine Research in Oxford, sally.beer@ouh.nhs.uk  **Alexis Espinosa**, Senior Research Nurse, Emergency Medicine Research in Oxford, alexis.espinosa@ouh.nhs.uk  **Giles Bond Smith**, Consultant in General Surgery, Oxford University Hospitals NHS Foundation, giles.bond-smith@ouh.nhs.uk  **Cyrene Saga**, Senior Radiographer, Oxford University Hospitals NHS Foundation, cyrene.saga@ouh.nhs.uk  **Jane Andrews**, Neuroradiology Manager, MRI Modality Lead, Oxford University Hospitals NHS Foundation, jane.andrews@ouh.nhs.uk  **Dr Adam Bailey**, Consultant in Gastroenterology, Oxford University Hospitals NHS Foundation Trust, adam.bailey@ouh.nhs.uk  **Mr Zahir Soonawalla**, Consultant Surgeon, Oxford University Hospitals NHS Foundation Trust, zahir.soonawalla@ouh.nhs.uk  **Dr Helen Bungay**, Consultant Radiologist, Oxford University Hospitals NHS Foundation Trust, https://orcid.org/0000-0003-1016-3121, helen.bungay@ouh.nhs.uk  **Professor Michael Pavlides**, Consultant in Hepatology, Oxford University Hospitals NHS Foundation Trust; Head of Liver Imaging Research, Oxford Centre for Clinical Magnetic Resonance Research (OCMR), University of Oxford; Associate Professor of Hepatology, Radcliffe Department of Medicine, University of Oxford, michael.pavlides@ouh.nhs.uk, https://orcid.org/0000-0001-9882-8874 |

**Corresponding author**

Professor Alex Novak, alex.novak@ouh.nhs.uk, https://orcid.org/0000-0002-5091-6768

Consultant in Emergency Medicine and Ambulatory Care, Emergency Medicine Research Oxford (EMROx), Oxford University Hospitals NHS Foundation Trust

Address: John Radcliffe Hospital, Emergency Department, Headley Way, Headington, Oxford OX3 9DU

**Supplementary Tables**

***Supplementary Table 1:*** *Breakdown of the screening and enrolment log. Total number screened 305.*

|  |  |  |
| --- | --- | --- |
| **Screening and enrolment log** | | **Number of patients** |
| **ED referral to SEU** | | |
|  | Recruited | 1 |
|  | Not recruited as USS already done | 1 |
|  | **Total** | **2** |
| **SEU admissions** | | |
|  | Excluded patients | 126 |
|  | Declined | 14 |
|  | Unable to consent | 3 |
|  | USS done before approach | 61 |
|  | CT done before approach | 19 |
|  | MRI done before-approach | 13 |
|  | MRI-no capacity | 42 |
|  | Enrolled | 27 |
|  | **Total** | **303** |

**Supplementary Table 2:** Patient demographics

|  | **Standard Care**  n = 11 | **Intervention**  n = 14 | **Total**  n = 26 |
| --- | --- | --- | --- |
| Age (years) | 46 (19-77) | 52 (23-73) | 50.5 (19-77) |
| Gender (% female) | 7 (64) | 11(73) | 18 (69) |
| Gender (% male) | 4 (36) | 4 (27) | 8 (31) |
| Presenting complaint abdominal pain (%) | 9 (82) | 15 (100) | 29 (92) |
| Presenting complaint chest pain (%) | 2 (18) | 0 (0) | 2 (8) |
| Emergency Department presentation (ED) (%) | 10 (91) | 10 (67) | 20 (77) |
| Surgical Emergency Unit presentation (SEU) (%) | 1 (9) | 4 (27) | 5 (19) |
| Ambulatory Assessment Unit presentation (AAU) (%) | 0 (0) | 1 (7) | 1 (4) |

***Supplementary Table 3:*** *Patient reported symptoms upon enrolment to the study.*

| **Characteristic** | **Intervention**,  N = 9 | **Standard of Care**, N = 8 | **p-value** |
| --- | --- | --- | --- |
| ***Symptoms*** |  |  |  |
| Abdominal pain | 9 (100%) | 8 (100%) | >0.99 |
| Chest pain | 2 (22%) | 3 (38%) | 0.62 |
| Back pain | 1 (11%) | 3 (38%) | 0.29 |
| Vomiting | 2 (22%) | 4 (50%) | 0.34 |
| Nausea | 1 (11%) | 3 (38%) | 0.29 |
| Diarrhoea | 0 (0%) | 1 (12%) | 0.47 |
| None | 9 (100%) | 8 (100%) | >0.99 |
| ***Duration of symptoms*** |  |  | 0.21 |
| Less than 24 hours | 7 (78%) | 3 (38%) |  |
| A week or less | 1 (11%) | 4 (50%) |  |
| A month or less | 0 (0%) | 0 (0%) |  |
| A year or less | 0 (0%) | 0 (0%) |  |
| More than a year | 1 (11%) | 1 (12%) |  |
| ***Frequency of symptoms*** |  |  | **0.04** |
| Daily | 2 (22%) | 1 (12%) |  |
| Once a week | 0 (0%) | 3 (38%) |  |
| Every week | 0 (0%) | 2 (25%) |  |
| Every month | 7 (78%) | 2 (25%) |  |
| ***Impact of symptoms*** |  |  | 0.47 |
| A huge amount | 1 (11%) | 3 (38%) |  |
| A lot | 2 (22%) | 2 (25%) |  |
| A fair amount | 2 (22%) | 1 (12%) |  |
| A little | 4 (44%) | 1 (12%) |  |
| Not much | 0 (0%) | 1 (12%) |  |
| ***Severity of symptoms*** | 8 (7, 10) | 9 (8, 10) | 0.54 |

***Supplementary Table 4:*** *Patient reported satisfaction with treatment*.

| **Characteristic** | **Intervention**,  N = 9 | **Standard of Care**, N = 8 | **p-value** |
| --- | --- | --- | --- |
| ***Satisfaction with time to diagnosis*** |  |  | >0.99 |
| Very satisfied | 4 (44%) | 3 (38%) |  |
| Satisfied | 4 (44%) | 3 (38%) |  |
| Neither satisfied nor unsatisfied | 0 (0%) | 0 (0%) |  |
| Unsatisfied | 1 (11%) | 1 (12%) |  |
| Very unsatisfied | 0 (0%) | 1 (12%) |  |
| ***Satisfaction with treatment of symptoms*** |  |  | 0.38 |
| Very satisfied | 5 (56%) | 2 (25%) |  |
| Satisfied | 4 (44%) | 4 (50%) |  |
| Neither satisfied nor unsatisfied | 0 (0%) | 1 (12%) |  |
| Unsatisfied | 0 (0%) | 1 (12%) |  |
| Very unsatisfied | 0 (0%) | 0 (0%) |  |
| ***Satisfaction with treatment of disease*** |  |  | 0.65 |
| Very satisfied | 3 (33%) | 3 (38%) |  |
| Satisfied | 5 (56%) | 2 (25%) |  |
| Neither satisfied nor unsatisfied | 0 (0%) | 1 (12%) |  |
| Unsatisfied | 1 (11%) | 1 (12%) |  |
| Very unsatisfied | 0 (0%) | 1 (12%) |  |

***Supplementary Table 5:*** *Patient reported symptoms in the three months after enrolment.*

| **Characteristic** | **Intervention**,  N = 9 | **Standard of Care**, N = 8 | **p-value** |
| --- | --- | --- | --- |
| ***Reported symptoms requiring GP visit*** |  |  |  |
| Abdominal pain | 1 (11%) | 1 (12%) | >0.99 |
| Chest pain | 0 (0%) | 0 (0%) | >0.99 |
| Back pain | 0 (0%) | 0 (0%) | >0.99 |
| Vomiting | 1 (11%) | 0 (0%) | >0.99 |
| Nausea | 0 (0%) | 0 (0%) | >0.99 |
| Diarrhoea | 0 (0%) | 0 (0%) | >0.99 |
| Other | 0 (0%) | 1 (12%) | 0.47 |
| None | 7 (100%) | 6 (0%) | >0.99 |
| ***Reported symptoms requiring hospital visit*** |  |  |  |
| Abdominal pain | 0 (0%) | 1 (12%) | 0.47 |
| Chest pain | 0 (0%) | 0 (0%) | >0.99 |
| Back pain | 0 (0%) | 0 (0%) | >0.99 |
| Vomiting | 0 (0%) | 0 (0%) | >0.99 |
| Nausea | 0 (0%) | 0 (0%) | >0.99 |
| Diarrhoea | 0 (0%) | 0 (0%) | >0.99 |
| Other | 0 (0%) | 0 (0%) | >0.99 |
| None | 9 (100%) | 7 (88%) | 0.47 |

**Supplementary Figures**

***Supplementary figure 1:*** *Groupwise differences in MRCP+ metrics between those with and without gallstones*


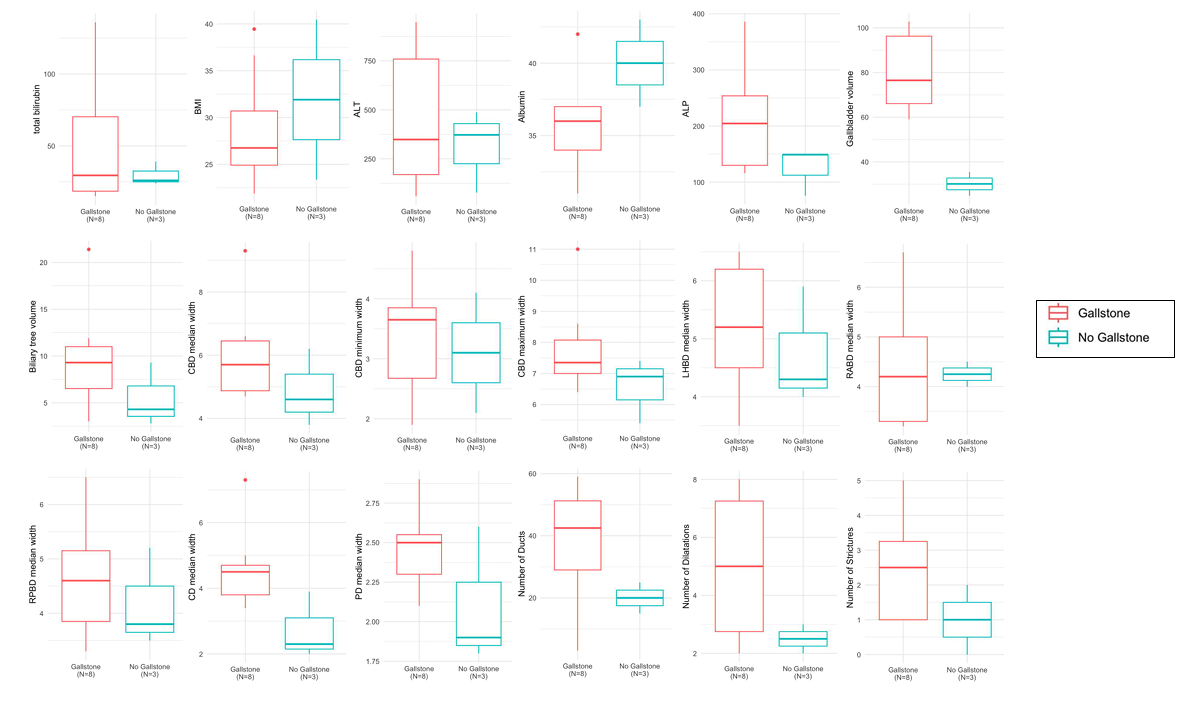


***Supplementary figure 2:*** *Example MRCP images for patients with suspected acute gallstone disease who were later diagnosed with a) no gallstones, b) stones within the gallbladder, and c) stones within the common bile duct (CBD). Corresponding quantitative MRCP+ models for each patient are also shown (d-f).*


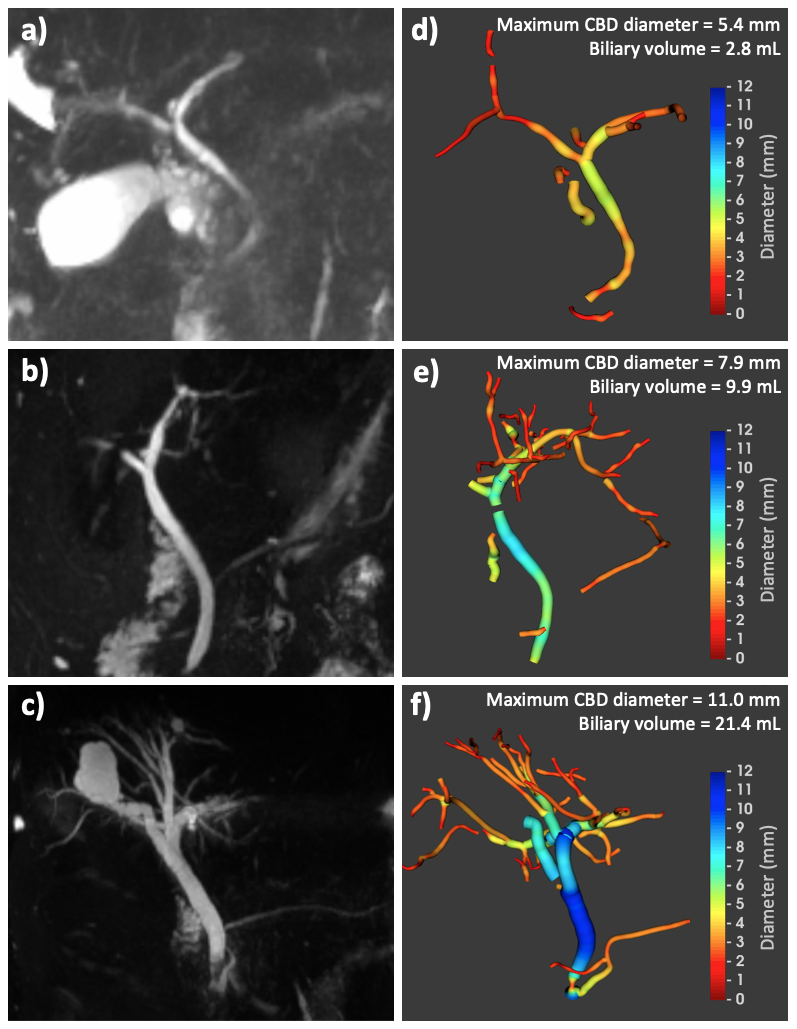

Supplement: Supplementary file 1 — Supplementary Material 1 [file 12876_2025_3637_MOESM1_ESM.docx]
